# Evaluating a Novel Infant Heart Rate Detector for Neonatal Resuscitation Efforts: Protocol for a Proof-of-Concept Study

**DOI:** 10.2196/45512

**Published:** 2023-10-02

**Authors:** Abdelrahman Abdou, Sridhar Krishnan, Niraj Mistry

**Affiliations:** 1 Department of Electrical, Computer and Biomedical Engineering Toronto Metropolitan University Toronto, ON Canada; 2 Scarborough Health Network Toronto, ON Canada

**Keywords:** newborn, electrocardiogram, ECG, dry electrode, heart rate, pediatric, resuscitation, infant, vital signs, neonatal

## Abstract

**Background:**

Over 10 million newborns worldwide undergo resuscitation at birth each year. Pediatricians may use electrocardiogram (ECG), pulse oximetry (PO), and stethoscope in determining heart rate (HR), as HR guides the need for and steps of resuscitation. HR must be obtained quickly and accurately. Unfortunately, the current diagnostic modalities are either too slow, obtaining HR in more than a minute, or inaccurate. With time constraints, a reliable robust heart rate detector (HRD) modality is required. This paper discusses a protocol for conducting a methods-based comparison study to determine the HR accuracy of a novel real-time HRD based on 3D-printed dry-electrode single-lead ECG signals for cost-effective and quick HR determination. The HRD’s HR results are compared to either clinical-grade ECG or PO monitors to ensure robustness and accuracy.

**Objective:**

The purpose of this study is to design and examine the feasibility of a proof-of-concept HRD that quickly obtains HR using biocompatible 3D-printed dry electrodes for single-lead neonatal ECG acquisition. This study uses a novel HRD and compares it to the gold-standard 3-lead clinical ECG or PO in a hospital setting.

**Methods:**

A cross-sectional study is planned to be conducted in the neonatal intensive care unit or postpartum unit of a large community teaching hospital in Toronto, Canada, from June 2023 to June 2024. In total, 50 newborns will be recruited for this study. The HRD and an ECG or PO monitor will be video recorded using a digital camera concurrently for 3 minutes for each newborn. Hardware-based signal processing and patent-pending embedded algorithm-based HR estimation techniques are applied directly to the raw collected single-lead ECG and displayed on the HRD in real time during video recordings. These data will be annotated and compared to the ECG or PO readings at the same points in time. Accuracy, *F*_1_-score, and other statistical metrics will be produced to determine the HRD’s feasibility in providing reliable HR.

**Results:**

The study is ongoing. The projected end date for data collection is around July 2024.

**Conclusions:**

The study will compare the novel patent-pending 3D-printed dry electrode–based HRD’s real-time HR estimation techniques with the state-of-the-art clinical-grade ECG or PO monitors for HR accuracy and examines how fast the HRD provides reliable HR. The study will further provide recommendations and important improvements that can be made to implement the HRD for clinical applications, especially in neonatal resuscitation efforts. This work can be seen as a stepping stone in the development of robust dry-electrode single-lead ECG devices for HR estimations in the pediatric population.

**International Registered Report Identifier (IRRID):**

DERR1-10.2196/45512

## Introduction

### Background

Each year, over 10 million newborns require resuscitation worldwide, representing more than 10% of all global births [1]. Neonatal resuscitation is the set of interventions at the time of birth to support the establishment of breathing and circulation. These interventions include providing warmth to the infant using a heat source, placing the head in a sniffing posture with neck flexion and upper cervical extension (a position to open the airways), drying the infant, and simulating breathing. Multiple metrics—respiration, infant color, and heart rate (HR)—are measured every 30 seconds to ensure successful neonatal resuscitation [2]. Out of these indicators, HR is identified as the most important for an effective resuscitation process [3]. HR guides decision-making and escalation of care, including positive pressure ventilation, endotracheal intubation, chest compressions, and administration of epinephrine.

Despite the importance of HR in neonatal resuscitation, HR determination is challenging due to a lack of evidence regarding the most rapid and accurate method to assess it. Clinically accepted methods of HR assessment include pulse palpation (umbilical, brachial, and femoral), auscultation with a stethoscope, pulse oximetry (PO) monitoring, and 3-lead electrocardiogram (ECG) monitoring [2]. It is important to note that neonatal resuscitation efforts should be performed within the first minute of life, known as the “golden minute,” to ensure a high success rate and avoid neurodevelopmental disorders and hypoxic injury [4].

A systematic review compared these different methods in terms of expense, intermittent or continuous HR assessment, and other strengths and weaknesses. Auscultation and pulse palpation are rapid and easily accessible approaches due to their low cost. However, both approaches are highly inaccurate and unreliable, resulting in errors of omission, which causes failure or delay in resuscitation steps and commission through inappropriate intervention [5]. Auscultation requires a pause in resuscitative efforts, which can disrupt the flow of resuscitation and result in a delay in stabilization of the newborn. This method is the most used approach compared to other HR determination approaches, due to its quick and easy accessibility as well as its cost-effectiveness. However, auscultation accuracy is reliant on health care professional experience and can be inconsistent from one practitioner to the other, resulting in inequitable care [6]. On the other hand, PO is relatively inexpensive and noninvasive, and it displays continuous HR and oxygen saturation to the entire team in the delivery room. However, a pulse oximeter can be inaccurate due to motion artifact, ambient light, or poor tissue perfusion, and it takes several minutes to apply and achieve the first data display, potentially delaying resuscitative efforts [4,5].

Current guidelines suggest a 3-lead ECG monitor as the gold standard for HR determination in neonates due to its high speed and accuracy and because of its capabilities to provide continuous visual and auditory data compared to the other modalities, such as PO and auscultation. However, 3-lead ECG has its disadvantages. Errors in the placement of the 3 leads can yield inappropriate ECG signals that cannot be used for HR detection [7]. Additionally, similar to PO, wet electrode ECG placement takes time to apply, surpassing the golden minute of life [5]. ECG adhesive wet electrodes can cause skin damage to premature infants. Moreover, motion artifacts may be present with ECG leads. In rare scenarios, ECG-derived HR may fail to correspond with the effective mechanical contraction of the heart [8]. Most importantly, both PO and 3-lead ECG require additional equipment, which needs to be purchased and maintained, accumulating significant expenses for health care systems [7,8]. Both technologies may not be feasible or accessible in areas with limited health care resources [9].

Recent rapid scientific advancement in sensing technology have driven the development of novel technologies to assess HR, including camera-based photoplethysmography, reflectance PO, laser Doppler technology, capacitive sensors, piezoelectric sensors, electromyography, and electronic or digital stethoscope (DS) [10]. Although a systematic review of these novel technologies concluded that none are ready for widespread clinical adoption, DS offers the greatest potential [10]. Currently, DS is at the forefront in the field of “computer-aided auscultation,” as advances in acoustic sensor design, digital signal processing and filtering, auditory enhancement, and machine learning can enable automated diagnoses of cardiac dysfunction [10,11]. In neonates, DS used for HR measurement is more accurate than auscultation or pulse palpation and faster than PO in “time to first HR display”; DS also correlates appropriately with ECG HR in noncrying infants [10-12]. Furthermore, DS is most similar to existing smart device technologies that are rapidly adopted, in both high- and low-resource settings. These technologies include smartphones, wearables, and other devices in the Internet of Medical Things. Although DS represents a significant improvement in neonatal resuscitation HR monitoring, further refinement and evaluation is necessary before clinical implementation, along with optimization of the existing technologies in a user-centric manner [13]. DS has severe disadvantages, including short battery life, low clinical adoption due to training requirements [14], and its nonstandardized variable sensor technologies, such as the differences between Littman Eko DUO with an ECG module, Littman CORE with analog and digital amplification, and Thinklabs’ One DS with a nonstandard headphone jack. It is important to note that none of the current DS technologies take neonatal ECG into consideration during their development.

It is evident there is a lack of cost-effective and fast signal acquisition technology that uses the gold-standard ECG for quick HR measurements and display in newborns. This study focuses on the deployment of a single-lead ECG handheld monitor capable of using dry-electrode ECG for signal acquisition and HR measurements in a quick and efficient manner. The following protocol study is structured as follows: the study significance, its impact on the current technologies in single-lead ECG devices for neonatal HR monitoring, and the objectives of this study. The Methods section is presented next, and it covers an in-depth look into the ethics-approved protocols through Study Design, Study Personnel, Recruitment, Inclusion and Exclusion Criteria, Ethical Considerations, and Study Procedures sections. Study Procedures section includes the subsections of Data Collection, Debrief, Data Management, Data Analysis, and Data Maintenance and Security. These sections are followed by the Results section, which offers the current progress of this study. Lastly, the final section provides a brief discussion of the protocol and the limitations of the current study, providing future steps to further improve neonatal single-lead ECG monitoring.

### Study Significance and Impact

Given that no single method of HR assessment is clearly superior to others, finding the optimal method to delineate HR within the golden minute after birth remains elusive. Pediatric guidelines recognize this knowledge gap and identify the opportunity to develop new technologies for the rapid, accurate assessment of HR after birth, ideally integrating newborn ECG. Currently, there are no novel medical devices that have been specifically developed to provide rapid and accurate HR immediately after birth. The proposed research study will address several gaps in the scientific study of device development, vital signs measurement and monitoring, and neonatal resuscitation. Recent advances in biomedical sensors and wireless communication technologies have enabled us to develop a novel HRD, targeted to outperform the traditional HR assessment in neonatal resuscitation. The rapid application, measurement, and display of HR on a device located on the neonate addresses significant gaps in the provision of optimal neonatal resuscitation. The HRD is undergoing rigorous design and development, and this study aims to assess its feasibility in quickly detecting accurate HRs in infants using 3D-printed dry electrodes. This early proof-of-concept HRD study will establish the basis for using 3D-printed dry electrodes and determine whether novel HR estimation algorithms are appropriate for neonatal HR applications.

### Objectives

The overall aim of this research study is to evaluate a novel HRD that may enable timely and appropriate neonatal resuscitation. This study is being completed using a phased sequential user-centered approach. We completed the first phase of this research to determine end-user requirements and preferences for HR assessment in neonatal resuscitation. This phase resulted in the current design and features of the HRD.

The second phase outlines the creation of the HRD conceptual design and proof-of-concept prototype to be used in the final phase of feasibility mentioned in this study.

In the final phase, the focus of this proof-of-concept research study is to examine the functionality, speed, robustness, and reliability of the prototype HRD.

Specific research questions to be answered through this phase are the following:

- How long does it take to place the HRD on an infant?
- Can the prototype HRD detect the HR of newborns?
- How fast can the prototype detect the HR of newborns?
- How accurate is the HR detection when compared to PO or ECG?

## Methods

### Study Design

**Figure 1:**
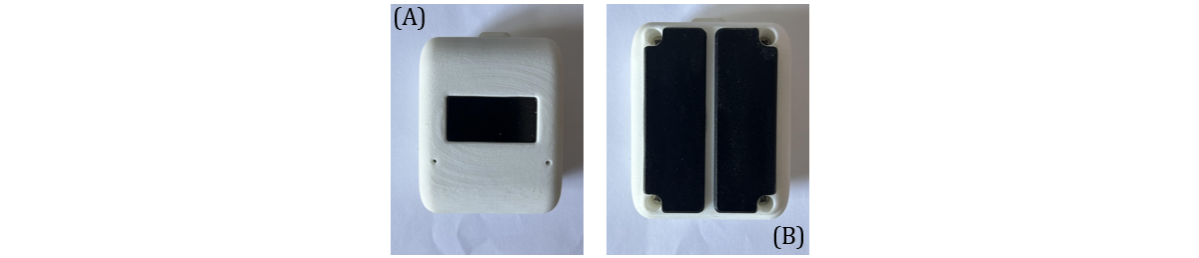
Heart rate detector (HRD) prototype. (A) The front side of the HRD with an OLED display to present heart rate in real time; (B) the backside of the HRD with two 3D-printed dry electrodes for single-lead neonatal electrocardiogram acquisition.

**Figure 2:**
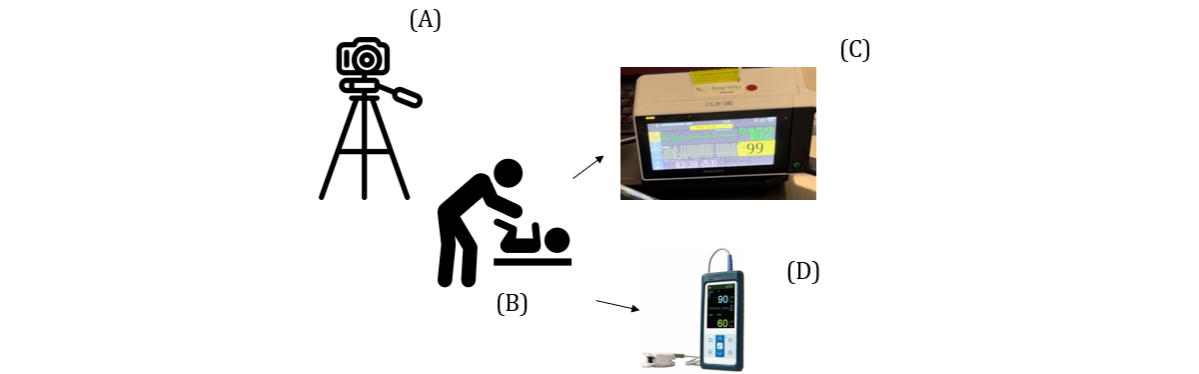
Data collection setup. (A) A digital camera is used for 3-minute video recordings; (B) a pediatrician holding the heart rate detector (HRD) to the newborn’s thorax; (C) electrocardiogram monitor and (D) pulse oximetry, present in the video recordings with HRD for comparison.

A prospective observational study will be used to measure the performance of the HRD prototype, as shown in Figure 1, in terms of time required to place it on an infant, its ability to detect HR, the speed of detection, and its reliability (accuracy) in comparison with clinical HR monitors. Given the paucity of data on infants regarding the HRD, there is insufficient evidence to support the feasibility and performance of a randomized controlled trial. The study setting will be in the postpartum unit (PPU) and neonatal intensive care unit (NICU) of a large community teaching hospital in Toronto, Canada. Specifically, the study will take place within 2 physical settings at the hospital: PPU and NICU. As shown in Figure 2, a digital camera will be used to record the HRD concurrently with either an ECG or PO monitor, depending on their availability at the time, for 3-minute durations per newborn. It is important to note that although both 3-lead ECG or photoplethysmogram technology are compared to single-lead dry-electrode ECG, both clinical devices are considered acceptable for neonatal resuscitation efforts, which is the targeted use of the HRD in later versions and research studies.

**Figure 3:**
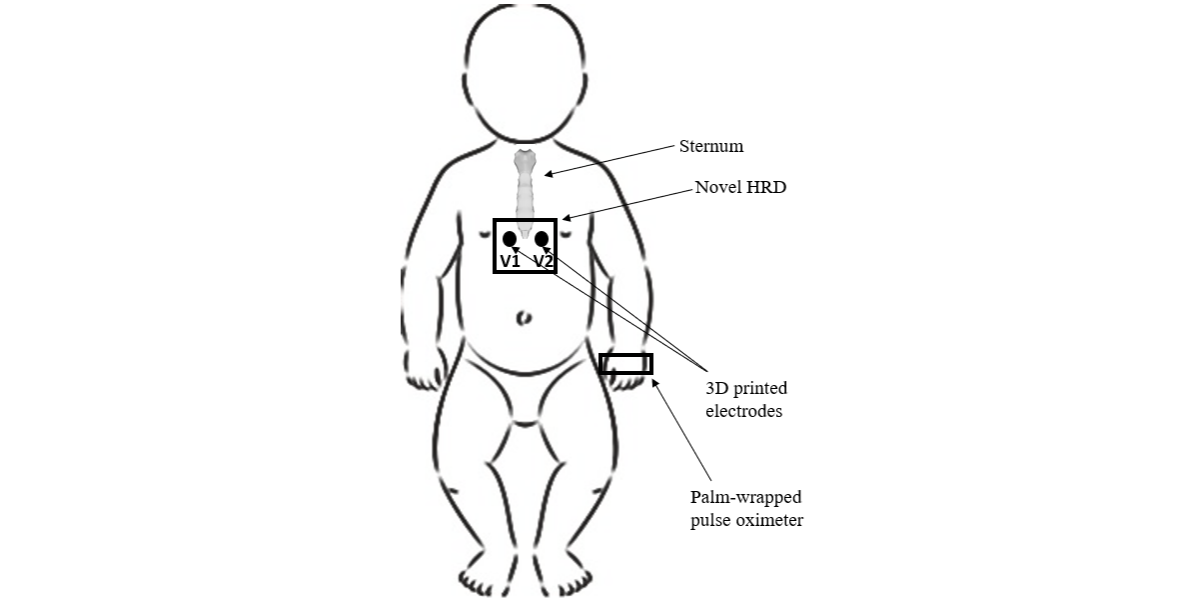
The placement of heart rate detector’s dry electrodes on the newborn's chest. HRD: heart rate detector.

**Figure 4:**
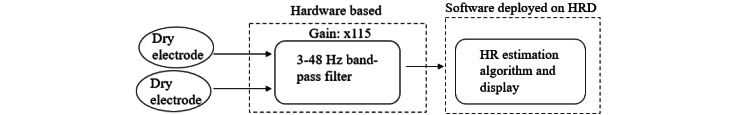
The hardware and software components of the heart rate detector (HRD).

The HRD uses two 3D-printed dry electrodes to collect 3-minute single-lead neonatal ECG signals with a 500 Hz sampling rate. The HRD’s electrodes are placed approximately in the V1 and V2 locations on the newborn’s chest, as shown in Figure 3. The electrodes are developed using conductive polylactic acid filaments (Proto Pasta) and a 3D printer with printing settings of nozzle temperature at 215 °C, print speed of 35 mm/s, heated bed temperature of 60°C, and a design fill ratio of 100% [15]. These settings are chosen as specified under the datasheets provided for the conductive polylactic acid filament. As shown in Figure 4, the electrodes are connected to an ECG analog front-end circuitry, which provides analog signal processing and filtering. A 3-48 Hz analog bandpass filter is applied to attenuate the powerline and electromagnetic interference noise in the 50-60 Hz range [15]. The single-lead ECG signal is later transmitted to a microcontroller, where patent-pending embedded heart estimation techniques are used to calculate HR. HR is displayed on an OLED screen on the HRD. At the same time, the HR and raw ECG information are transmitted using Bluetooth to a nearby computer interface for data storage.

### Study Personnel

A pediatrician and graduate student will be conducting all the study tasks.

### Recruitment

All Infants admitted to the PPU after routine delivery, regardless of method, and all infants admitted to the NICU will be eligible if their clinical status is stable and not at high risk of deterioration. In total, 50 newborns will be recruited based on previous accepted research studies that focused on examining long-term neonatal ECG and HR monitoring applications using proof-of-concept novel ECG acquisition devices [14-19].

The two groups of infants are identified as follows:

1. Infants admitted to the PPU who are at least 24 hours of age and are undergoing routine critical congenital heart disease (CCHD) screening with PO.
2. Infants admitted to the NICU who are routinely placed on 3-lead ECG or PO monitors.

Recruitment steps include the following:

Infants will be identified by a pediatrician for eligibility 5 days a week (Monday to Friday) between 8 AM and 8 PM. Recruitment on Saturday and Sunday will be done if feasible. Recruitment will happen on random days when a pediatrician is available and time permits.

Once an infant is identified based on eligibility (ie, inclusion and exclusion criteria), mentioned below, the pediatrician will ask the bedside nurse to ask the parents if they would be willing to speak to the pediatrician as shown in the recruitment script in Multimedia Appendix 1.

The pediatrician will then meet the parents in a face-to-face meeting to discuss the study, provide the written consent form, and potentially obtain consent. The parents will be given at least 15 minutes to review the consent form prior to being asked to sign it. When the parents are not available at the bedside, the pediatrician will inquire the nurse about a suitable time for the parents to meet. The infants will not be under the care of the researcher pediatrician as their primary admitting health practitioner to avoid a conflict of interest.

#### Inclusion Criteria

The inclusion criteria for the PPU infants are as follows:

- Corrected gestational age (35 weeks + 0 days)
- Infants undergoing routine CCHD screening with PO at 24 hours of life or later
- Stable clinical status
- Parental consent

For the NICU infants, the inclusion criteria are as follows:

- Corrected gestational age (30 weeks + 0 days)
- Infants undergoing routine 3-lead continuous ECG monitoring or PO
- Infants with at least 24 hours of life
- Stable clinical status, as defined by the following: stable requirement for supplemental oxygen and stable respiratory rate on at least 2 measurements, a respiratory rate <70 breaths per minute, HR <180 beats per minute, oxygen supplementation <0.5 L/min via nasal prongs, and not being on heated high-flow oxygen
- Parental consent

#### Exclusion Criteria

The exclusion criteria include high-risk clinically unstable newborns who might show unstable requirements for supplemental oxygen and unstable respiratory rates, congenital malformations that interfere with the placement of the HRD, and nonconsent.

The criteria mentioned above are standardized points used in multiple studies pertaining to neonatal noninvasive physiological monitoring. These criteria have undergone rigorous ethics review, as specified in the approved ethics proposals.

### Ethical Considerations

The study was approved by the research ethics boards of the Toronto Metropolitan University (2023-070) and Scarborough Health Network (PED-21-025) in April and June of 2023, respectively. Parents will complete a detailed written informed consent prior to infant enrollment. Recruitment procedures are described under the Recruitment subsection in Methods. A number of measures will be taken to protect the rights of eligible infants, including the following: (1) using an intermediate approach—a neutral person, a bedside postpartum or NICU nurse, will seek initial permission to approach potential participants about the study; (2) ensuring anonymity, privacy and confidentiality of the participant’s information; and (3) proceeding through the formal ethical review process at both the participating hospital and the university conducting the collaborative research. As per institutional policies, consent forms will be kept in a secured location, with access provided to only the primary principal investigator and then shredded. Finally, participants will be assured that the study reports, video recordings, and paper-based data collection forms will not contain identifying information in keeping with the Canadian privacy legislations—Personal Information Protection and Electronic Documents Act and Personal Health Information Protection Act.

### Study Procedures

The PPU infants will be placed in the supine (face up) position. The conventional PO will be connected to the right hand as per the device’s instructions and CCHD screening protocol. In the usual standard of care for CCHD screening, PO is placed on the right hand and right or left foot for 1 to 2 minutes. The change in the standard of care will include the data collection protocol during the time of CCHD screening, which includes video recording of the HRD and PO simultaneously. The HRD will be held in place by a pediatrician on the thorax for 3 minutes. This will prolong CCHD screening by an additional 1-3 minutes. On the other hand, as per the usual standard of care, NICU infants will be placed in the supine (face up) position prior to their usual feeding time; they will be unbundled and have their diaper changed. An already attached conventional 3-lead ECG will be placed on the infant’s chest, as per the conventional 3-lead ECG device’s instructions. The change in this standard of care will involve the thorax to be exposed, if not already exposed, and the HRD will be held in place by a pediatrician on the thorax for 3 minutes during simultaneous video recording of the HRD and the 3-lead ECG monitor. It is important to note that the 3-lead ECG electrodes will not be in contact with the HRD’s dry electrodes.

The displayed HR from the HRD will be compared to 1 or 2 reference monitors, depending on the infant’s clinical setting (PPU or NICU). The PPU uses a conventional PO (Nellcor Portable SpO2 Patient Monitoring System; Medtronic) with an accompanying sensor wrap, while infants in the NICU are attached to a conventional 3-lead gel electrode ECG (Intellivue Bedside Patient Monitor; Koninklijke Philips) monitor. In both clinical-grade devices, PO and ECG biosensors and algorithms detect and display HR based on pulse peak or QRS peak detection. An internal algorithm in each device designed by the manufacturer determines how pulse or QRS peak detection is transformed into the HR displayed on the screen. Depending on the sensing technology—PO (a light-emitting diode-based pulse detection) or the 3-lead ECG (an electric activity sensing transducer that detects electrical heart activity)—a different HR may be displayed. Differences in the PO and ECG algorithmic HR estimation techniques may also influence the HR displayed on both devices. However, both technologies are known as clinical-grade HRDs and are used extensively in medical settings, which justifies their use as the ground truth state in this HRD proof-of-concept study.

#### Data Collection

The displayed HR from the HRD and the PO or ECG monitor will be video recorded for 3 minutes within the same video frame, as shown in Figure 1. Video recording will take place using a dedicated research digital camera mounted on a stand. Video recording will start once the infant is settled and the PO or ECG readings are stable. Stable readings are defined based on the device’s instructions on waiting for an appropriate amount of time after sensor placement for clinical HR determination. The HRD will then be placed on the infant’s chest until a consistent HR is displayed for 3 minutes, at which point video recording will start. After 3 minutes, the HRD will be removed, and then, the recording will be stopped. After the recording is completed, the entire HRD, including electrodes, will be wiped down with a surface disinfectant towelette (CaviWipe; Metrex). HRD device failure will be considered if no HR is displayed on the HRD within 60 seconds of application to the thorax.

#### Debrief

After video recording has concluded, parents will be asked of their perceptions of the HRD, and their answers will be written by the primary researcher on a paper data collection form, as shown in Multimedia Appendix 2.

#### Data Management

Video recordings will be individually analyzed by 2 health professional researchers to determine the time it takes to apply the HRD to the thorax and the time from HRD placement to a steady HR display. Each researcher will review and record the HR from the HRD and PO or ECG at 2-second intervals for 3 minutes, providing approximately 90 HR notations per device per patient. For 50 newborns, the total number of HR annotations will be approximately 4500 data points. This will result in multiple HR data points to compare. To avoid human error in the annotation process, discrepancies in the HR of more than 5 beats per minute between the 2 researchers will lead to a reexamination of the video recordings until a consensus can be reached regarding the correct HR. The annotations from the 2 researchers will be averaged for the subsequent analyses.

Additionally, the HRD will record raw ECG signals for the duration of the device being in contact with the infant. Raw ECG data will be collected at 500 Hz sampling rate and transmitted to a research-dedicated laptop. The raw ECG and HR information from the HRD will be stored on different CSV files with a randomized identification number.

#### Data Analysis

HR and raw ECG data will be analyzed using MATLAB (the MathWorks). Infant demographics will be analyzed using summary statistics. Continuous variables will be expressed as mean (SD) values, and categorical variables will be expressed as numbers (percentages), unless otherwise stated. The HR from HRD will be compared to PO or ECG using a paired *t* test. The Bland-Altman method will be used to compare the HR derived from each device—the HRD and the PO or ECG monitor, used as reference. The correlation between devices will be assessed using Pearson *r* statistic. Data will be first analyzed using all data points available, and outliers will be defined as 20 beats per minute rate difference between the methods. These discrepancies will be further analyzed to find the likely reason for deviation, including reexamination of the video recordings.

#### Data Maintenance and Security

Each infant will be assigned a participant identification number using a random number generator [20]. No personally identifying demographic information, such as name or parents’ names, will be collected. Confidentiality will be maintained throughout the study, in accordance with the Personal Health Information Protection Act. Paper-based data collected will be electronically transcribed into a CSV file. The paper-based data collection form will then be disposed in a confidential document bin at the hospital. The data, video recordings, CSV files with raw ECG data, the CSV files with infant demographics and characteristics as well as parental perceptions will be downloaded to a research-dedicated laptop for analysis. The data will be stored in a password-protected folder and will not be emailed nor transmitted on the web. Data transfer among the research team (the primary researchers) will occur through an encrypted flash drive, if necessary. The laptop will be stored in a locked cabinet in a locked room in the research lab at the university, with access provided to only the primary research investigators. All the study investigators will have access to all the data, except the consent forms, which will be only accessible by the principal investigator.

## Results

The study is currently ongoing. The projected end date for data collection is around July 2024.

## Discussion

This paper discussed a research protocol for conducting accurate and reliable HR estimation using a novel real-time HRD based on 3D-printed dry-electrode single-lead ECG signals. Recent advances in biomedical sensor and wireless information and communication technologies have enabled us to develop a novel HRD, aimed at potentially disrupting the traditional assessment of HR in neonatal resuscitation efforts. The rapid application, measurement, and display of HR on a device mounted directly on the neonate addresses significant gaps in the provision of optimal neonatal resuscitation. The main objective of this study is to examine the novel HRD’s ability and feasibility in obtaining an accurate HR in newborns, using dry electrodes, and comparing it to the state-of-the-art clinical-grade HR monitors at the hospital.

However, this study is limited to the feasibility of HR detection on a healthy population of newborns using dry electrodes, and it does not cover in-depth analytical metrics of ECG and HR for disease diagnosis. It is apparent that further studies should focus solely on ECG abnormalities in neonates, including bradycardia, pulseless electrical activity, and long QT syndrome. The work is also limited to the use of certain 3D-printed conductive plastic-based dry electrodes for single-lead ECG acquisition, limiting its applicability when ECG quality is compared to other dry-electrode materials, such as stainless steel and textiles. Furthermore, the proposal does not include clinical-grade ECG acquisition for comparison with the HRD’s raw ECG signals due to the restraints in obtaining access to HR monitor raw ECG information. In addition, the use of algorithms on clinical-grade devices cannot be accessed, limiting this work to the examination of HR information between the HRD and clinical HR monitors. However, the raw ECG obtained through the HRD can be used to derive novel hand-crafted features that assist in quick HR estimation and prediction using artificial intelligence.

Ideally, this study will lead to critical improvements in the HRD prototype’s accuracy and speed of detection, ensuring its robustness prior to further clinical testing. This preliminary work is essential to form the foundation of a larger programmatic endeavor to transform this HRD into a relevant and robust novel medical device for neonatal resuscitation efforts. It is critical that new clinical technologies be evaluated with methodologic rigor to help inform clinicians and parents on the reliability, feasibility, and ultimately, the effectiveness of new technologies. This is an early proof-of-concept study to assess the reliability of the value behind this HRD and its goals of ultimately saving infant lives through widespread clinical adaptation and implementation.

## Appendix

Multimedia Appendix 1 Recruitment script.

Multimedia Appendix 2 Data collection form.

## Abbreviations

CCHD: critical congenital heart disease
DS: digital stethoscope
ECG: electrocardiogram
HR: heart rate
HRD: heart rate detector
NICU: neonatal intensive care unit
PO: pulse oximetry
PPU: postpartum unit

## Data Availability

The anonymized data pertaining to this study will be available upon request from the corresponding author, except for video recordings, which are not available in accordance with the ethics approvals of the participating hospital and academic institution.
