# Supplementary material for: Evaluating a Novel Infant Heart Rate Detector for Neonatal Resuscitation Efforts: Protocol for a Proof-of-Concept Study
Source: JMIR Res Protoc. 2023 Oct 2;12:e45512. doi: 10.2196/45512 (PMC10580137; doi:10.2196/45512)
Supplement: Multimedia Appendix 1 [file resprot_v12i1e45512_app1.pdf]

Appendix A - Recruitment Script

P = Potential Participant Parent; I = Initial Contact (Bedside Nurse)
I (PPU) – At this time/24 hours of life, all babies routinely get CCHD screening completed. One of our pediatricians, Dr. Mistry, is doing a research study that happens during the CCHD screening. Would you be willing to hear more about this research study?
P – Yes.
OR
P – No thank you.

I (NICU) – Your baby is on a ECG monitor. One of our pediatricians, Dr. Mistry, is doing a research study that happens while your baby is on this ECG monitor. Would you be willing to hear more about this research study?
P – Yes.
OR
P – No thank you.
