# Supplementary material for: Evaluating a Novel Infant Heart Rate Detector for Neonatal Resuscitation Efforts: Protocol for a Proof-of-Concept Study
Source: JMIR Res Protoc. 2023 Oct 2;12:e45512. doi: 10.2196/45512 (PMC10580137; doi:10.2196/45512)
Supplement: Multimedia Appendix 2 [file resprot_v12i1e45512_app2.pdf]

Appendix B – Data Collection Form

Infant Study Identification Number ____________

a. Age in hours, days or weeks

b. Gestational age and corrected gestational age

c. Birth weight and current weight

d. Sex

e. Medical problems for NICU infants including: oxygen requirement, nasogastric tube, venous access (IV or PICC line), chronic lung disease or congenital heart disease etc.

f. Parental perception of HRD – what are your thoughts about the HRD? What do you like about it? What do you not like about it?
